# MiR-218 produces anti-tumor effects on cervical cancer cells in vitro

**DOI:** 10.1186/s12957-018-1506-3

**Published:** 2018-10-12

**Authors:** Li Zhu, Huaidong Tu, Yanmei Liang, Dihong Tang

**Affiliations:** 1 Department of Gynecologic Oncology, The People’s Hospital of Taojiang County, Taojiang, China; 2 Department of Gynecologic Oncology, Hunan Cancer Hospital, No.283 Tongzipo Road, Yuelu District, Changsha, 410006 Hunan Province China

**Keywords:** MiR-218, Immune escape, Cervical cancer, IDO1, JAK2/STAT3, Apoptosis

## Abstract

**Background:**

As indoleamine-2,3-dioxygenase 1 (IDO1) is critical in tumor immune escape, we determined to study the regulatory mechanism of miR-218 on IDO1 in cervical cancer.

**Methods:**

Real-time PCR (RT-qPCR) was carried out to measure the expression of miR-218. RT-qPCR and Western blot were performed to detect the expression of IDO1 in cervical cancer. Dual-luciferase reporter assay was used to determine the binding of miR-218 on the IDO1 3′UTR. Cell viability, apoptosis, and related factors were determined using cell counting kit-8 (CCK-8), Annexin-V/PI (propidium) assay, enzyme-linked immunosorbnent assay (ELISA), RT-qPCR, and Western blot assays after miR-218 mimics has been transfected to HeLa cervical cancer cells.

**Results:**

MiR-218 was downregulated in cervical cancer. The expression of miR-218 was negatively correlated with IDO1 in cervical cancer tissues and cells. IDO1 is a direct target of miR-218. MiR-218 overexpression was found to inhibit cell viability and promoted apoptosis via activating the expression of Cleaved-Caspase-3 and to inhibit the expression of Survivin, immune factors (TGF-β, VEGF, IL-6, PGE2, COX-2), and JAK2/STAT3 pathway.

**Conclusion:**

MiR-218 inhibits immune escape of cervical cancer cells by direct downregulating IDO1.

## Background

Cervical cancer is one of the most common malignancies in the female reproductive system. Globally, the cancer has the second highest death rate among other cancer deaths among females [1, 2]. Advances in diagnostic technology allow many cervical cancer patients to be diagnosed and treated at an early stage [3, 4]. However, the mortality of cervical cancer is still high, accounting for approximately 50% [5, 6]. Studies have shown that tumor occurrence is closely related to the immune system, which has immune surveillance functions [7, 8]. The immune system can recognize and specifically eliminate “non-self” cells through immune mechanisms to resist the occurrence and development of tumors [9]. However, in some cases, via tumor immune escape process, malignant cells could result in occurrence, development, metastasis, and recurrence of tumors [10]. In addition, as the tumor grows, it can form a microenvironment that helps the tumor escape immune surveillance [11]. The tumor cells secrete immunosuppressive factors, to name a few, transforming growth factor-β(TGF-β). Interleukin-6 (IL-6), vascular endothelial growth factor (VEGF), prostaglandin 2 (PGE2), and cytochrome c oxidase subunit II (COX-2), etc., induce normal host cells to undergo immunosuppression, reduce immune function, and cause immune escape [12–15].

As a heme-containing enzyme, indoleamine-2,3-dioxygenase (IDO) is a negative immunoregulatory factor that not only decomposes tryptophan into multiple metabolites, therefore preventing an effective immune response of T cells, but also induces regulatory T cells (Tregs)-mediated immune escape [16, 17]. IDO was observed to be expressed in some primary tumors, for example, gastric cancer, colon cancer, and renal cell carcinoma [17, 18]. The expression of IDO1 in cervical cancer, breast cancer, ovarian cancer, endometrial cancer, colon cancer, and brain tumors predicts less satisfied clinical prognosis [19, 20]. In a study of non-small cell lung cancer, ovarian cancer and other tumor tissues, IDO1 was found to be associated with malignancy degrees of the tumors [21]. Studies have confirmed that IDO was overexpressed in primary myeloblasts, leading to a significant lack of tryptophan, and that IDO overexpression was also found to inhibit the proliferation of T cells, and this is because T cells in G1 phase are highly sensitive to tryptophan deficiency [19, 22]. On the other hand, the metabolites of tryptophan can cause apoptosis of T cells, leading to immune escape [23, 24]. Thus, inhibiting the proliferation of tumor cells by suppressing the function of IDO1 has drawn much research attention [25–27].

MicroRNA-218 (miR-218) is a tumor-suppressive miRNA in cancers. MiR-218 inhibits cell proliferation of glioma cells [28], osteogenic differentiation in synovial mesenchymal stem cells [29], and tumor angiogenesis in prostate cancer [30]. MiR-218 is downregulated in renal cell carcinoma (RCC) tissue, and cell proliferation is suppressed by miR-218 overexpression in RCC cells [31]. The bioinformatics analysis shows that IDO1 has binding targets of miR-218; we decided to study the mechanism of miR-218 functioning on IDO1 in the tumor immune escape of cervical cancer.

Therefore, we aimed to measure the effects of miR-218 on cervical cancer. This study will help to understand the principle of tumor development, to improve the immune status of the body, to reverse the tumor escape, and to design new treatment strategies.

## Methods

### Patients and tissues

Cervical cancer tissues were collected from The People’s Hospital of Taojiang County during October 2014 to January 2016. Patients with primary cervical squamous cell carcinoma and complete clinical data and who received radical hysterectomy and pelvic lymphadenectomy were included. The average age of all patients was 42.1 years old (22–63 years old). All patients did not receive radiotherapy or chemotherapy prior to surgery. Tissues were divided into different stages with reference to the International Federation of Gynecology and Obstetrics (FIGO) criteria. Clinical pathological features of patients with cervical cancer were shown in Table 1. The normal control was isolated from the corresponding adjacent non-carcinoma tissues of the patients with cervical cancer. The use of all tissue specimens was approved by the Ethics Committee of The People’s Hospital of Taojiang County. Tissues obtained were frozen and preserved in liquid nitrogen for mRNA analyses of miR-218 and IDO1. Spearman nonparametric correlation test was used to analyze correlation between miR-218 and IDO1.

**Table 1:** Clinical pathological features of patients with cervical cancer

Factors		IDO1 levels		*P* values
		Higher	Lower	
Age (years)	< 50	11	5	0.893
	≥ 50	14	7	
FIGO stages	I, II	7	10	0.002**
	III, IV	18	2	
Histological grade	Well/middle differentiated	8	8	0.046**
	Low differentiated	17	4	

**P < 0.01, chi-square test

### Cell culture and transfection

Human cervical epithelial cells (HcerEpic cells) and cervical cancer cells (HeLa, SiHa, C-33 and Caski cells) were purchased from Shanghai Institute of Cell Biology and cultured with Dulbecco’s modified Eagle’s medium (DMEM; Gibco, USA), which contained 10% fetal bovine serum (FBS; Gibco, USA), 1% penicillin, and streptomycin (Invitrogen, USA)with 5% CO_2_ at 37 °C. The cells cultured to logarithm phase were used in following experiments. The expression levels of miR-218 and IDO1 were first detected in above cell lines. The MiR-218 mimics (Mimics group) and NC control sequence (NC group) were synthesized by GenePharm (Shanghai, China) and then respectively transfected to HeLa cervical cancer cells using lipofectamine 2000 (Invitrogen, USA) as a transfection reagent. Cells with non-treatment were treated as control (Cntl group). Next, the expression levels of miR-218 and IDO1 were detected in Cntl, NC, and Mimics groups.

### Bioinformatics and dual-luciferase reporter assays

The potential target sequences of miR-218 in 3′-UTR fragment of IDO1 were predicted with reference to TargetScan website (http://www.targetscan.org/vert_72/). Next, a direct combination of miR-218 and IDO1 was verified by dual-luciferase reporter assay. Using the GeneTailor Site-Directed Mutagenesis System (Invitrogen, USA), the binding sequence of miR-218 on the 3′-UTR fragment of IDO1 was intentionally mutated. The IDO1-3′-UTR sequence or mutated IDO1-3′-UTR sequence (IDO1-3′-UTR mut) was then ligated to pmirGLO firefly and rinilla dual-luciferase reporter vector (Promega, USA). IDO1-3′-UTR or IDO1-3′-UTR mut recombinant luciferase reporter plasmid was co-transfected with miR-218 mimics. Finally, the luciferase activities were measured using the Dual-Glo™ Luciferase Reporter Assay System (Promega, USA) according to the manufacturer’s protocols.

### Cell counting kit-8 (CCK-8) assay

The effect of miR-218 mimics transfection on cell viability of HeLa cells was determined by CCK-8 assay (Beyotime, China). Cells in the Cntl, NC, and Mimics groups were respectively seeded into 96-well plates at a density of 5 × 10^3^ cells/well, with each experiments being repeated five times. After being incubated at 37 °C for 24 h, 20 μL CCK-8 reagent was added into each well for another 1 h of incubation at 37 °C. Next, optical density (OD) values were read at 450 nm using a microplate reader (Thermo, USA).

### Annexin-V/PI (propidium) assay

The effect of miR-218 mimics transfection on apoptosis of HeLa cells was determined by Annexin-V/PI assay (Roche, USA) according to the protocols of the manufacturer. Cells in Cntl, NC, and Mimics groups were respectively seeded in 6-well plates (5 × 10^4^ cells/well) and then put into reaction with 5 μl Annexin-V and 5 μl PI in the dark at 37 °Cfor 5 min. The apoptosis rates were analyzed using a flow cytometer (BD, USA) and Cell Quest software.

### Enzyme-linked immunosorbnent assay (ELISA)

The quantities of TGF-β, VEGF, IL-6, and PGE2 in the Cntl, NC, and Mimics groups were determined by ELISA kits (R&D, Minneapolis, USA) according to the manufacturer’s instructions. Samples were added into 96-well plate and incubated at 37 °C for 90 min, and biotinylated antibodies were then added into the plate and incubated for another 60 min. Next, avidin peroxidase complex (ABC) was added and incubated for 30 min prior to TMB (tetramethylbenzidine) coloration. Finally, OD values were read at 450 nm by a microplate reader (Thermo, USA), and the quantities were calculated by standard curve.

### Real-time-qPCR (RT-qPCR)

The mRNA levels of miR-218 and IDO1 were detected in cervical cancer tissues and cervical cancer cells (HeLa, SiHa, C-33, and Caski cells). In addition, the mRNA levels of miR-218, IDO1, Survivin, TGF-β, VEGF, IL-6, and COX-2 were detected in the Cntl, NC, and Mimics groups. The primers used were listed in Table 2. U6 (for miR-218) and GAPDH (for others) were used as internal control. Total RNA was extracted using Trizol reagent (Invitrogen, USA) and reversely transcribed to cDNA using Transcriptase (Roche, USA). The PCR amplification process was then conducted using LightCycler® Multiplex Masters (Roche, USA) with LightCycler® 480II System (Roche, USA). The procedures for miR-218 amplification were at 95 °C, 10 s, 40 cycles (at 95 °C, 10 s; at 60 °C, 30 s). The procedures for other target genes was at 95 °C, 5 min, 40 cycles (at 95 °C, 30 s, at 60 °C, 30 s, at 72 °C, 30 s) and at 72 °C, 10 min. The results were calculated using 2^−ΔΔCq^ method.

**Table 2:** The primer sequences applied in the study

Name	Type	Sequence (5′-3′)
GAPDH	Forward	CCATCTTCCAGGAGCGAGAT
	Reverse	TGCTGATGATCTTGAGGCTG
IDO1	Forward	GGGCTTTGCTCTACCACATCCACT
	Reverse	ACATCGTCATCCCCTCGGTTCC
Survivin	Forward	GGACCACCGCATCTCTACAT
	Reverse	TTGGTTTCCTTTGCATGGGG
TGF-β	Forward	TACAGCAACAATTCCTGGCG
	Reverse	GTGAACCCGTTGATGTCCAC
VEGF	Forward	CGGTATAAGTCCTGGAGCGT
	Reverse	TTTAACTCAAGCTGCCTCGC
IL-6	Forward	AGACAGCCACTCACCTCTTC
	Reverse	TTTCACCAGGCAAGTCTCCT
COX-2	Forward	ACCGTCTGAACTATCCTGCC
	Reverse	AGATTAGTCCGCCGTAGTCG
miR-218	Forward	TTGCGGATGGTTCCGTCA AGCA
	Reverse	ATCCAGTGCAGGGTCCGAGG
U6	Forward	AGAGAAGATTAGCATGGCCCCTG
	Reverse	ATCCAGTGCAGGGTC CGAGG

### Western blot

The protein levels of IDO1 were detected in cervical cancer tissues and cervical cancer cells (HeLa, SiHa, C-33, and Caski cells). In addition, the protein levels of IDO1, Survivin, Cleaved-caspase-3, Pro-caspase-3, TGF-β, VEGF, IL-6, COX-2, Janus kinase 2 (JAK2), phosphorylated-JAK2 (p-JAK2), signal transducers and activators of transcription 3 (STAT3), and phosphorylated-STAT3 (p-STAT3) were detected in the Cntl, NC, and Mimics groups. The proteins were first extracted by RIPA and quantified by BCA (Pierce, USA). Twelve percent of sodium dodecyl sulfate-polyacrylamide gel electrophoresis (SDS-PAGE) was used to isolate the proteins, which were then transferred to polyvinylidene fluoride (PVDF) membranes (ThermoFisher, USA). Next, the membranes were blocked in 5% non-fat dry milk for 1 h at 37 °C and incubated first with specific primary antibodies (Abcam, USA) overnight at 4 °C and then with secondary antibodies conjugated with horseradish peroxidase (CST, 7074, 1:5000, USA) at 37 °C for 1 h. GAPDH was used as loading control. The proteins were detected by enhanced chemiluminescense (ECL; Pierce, USA) and analyzed by Bio-Rad ChemiDoc XRS densitometry with Image Lab™ Software version 4.1 (Bio-Rad, USA).

### Statistical analysis

SPSS 18.0 statistical package with mean ± standard deviations (mean ± SD) was used to conduct statistical analysis. One-way analysis of variance (ANOVA) followed with Dunnett’s test was used to compare the differences. *P* < 0.05 was considered as significantly different.

## Results

### MiR-218 was downregulated and negatively correlated with IDO1 in cervical cancer tissues and cells

**Fig. 1:**
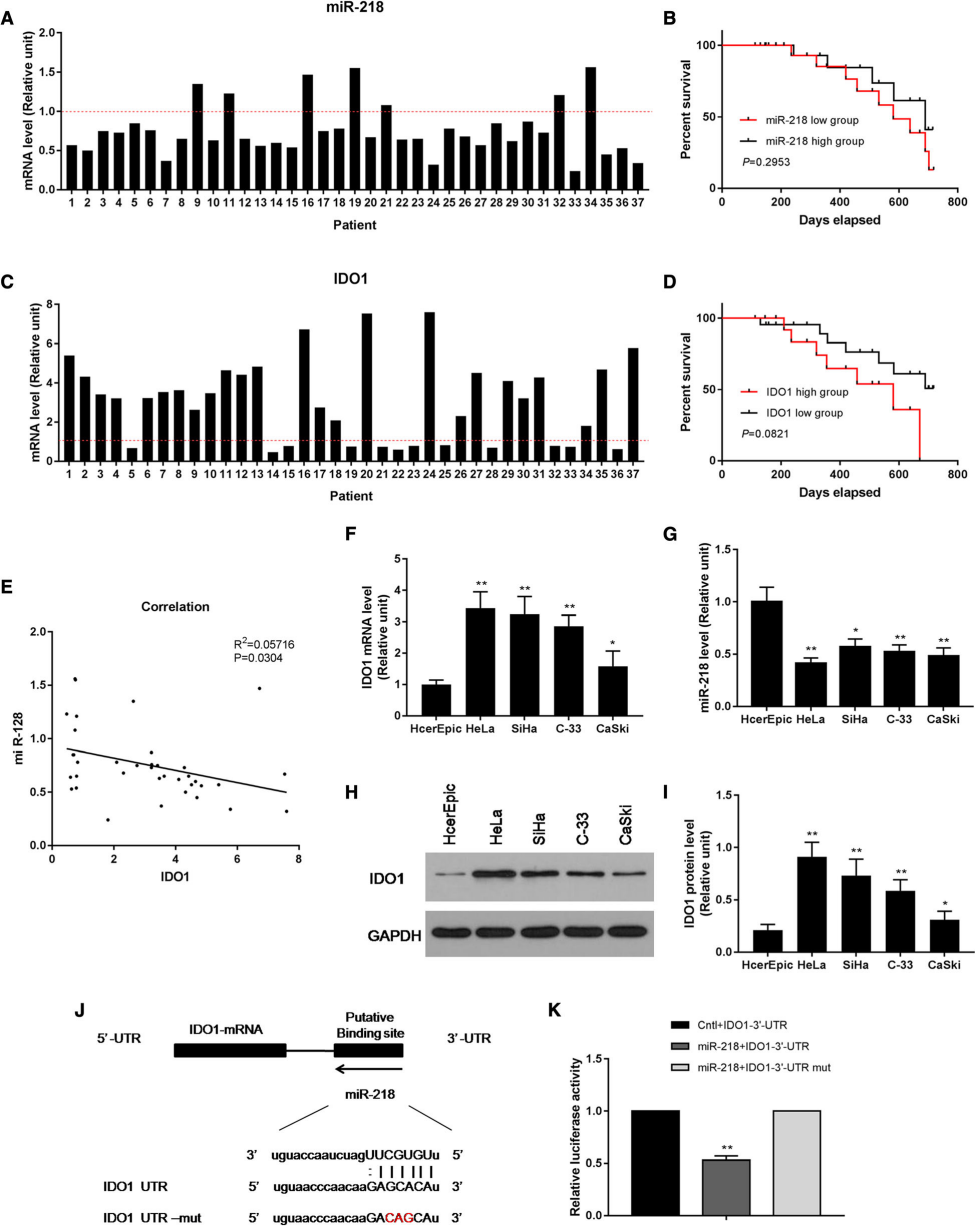
MiR-218 was upregulated and negatively correlated with IDO1 by direct targeting on IDO1 in cervical cancer tissues and cells. **a** The mRNA levels of miR-218 were downregulated in cervical cancer specimens. **b** The survival rate of miR-218 low group was lower than that in miR-218 high group. **c** The mRNA levels of IDO1 were upregulated in cervical cancer specimens. **d** The survival rate of IDO1 high group was lower than that in miR-218 high group. **e** MiR-218 and IDO1 were well negatively correlated in cervical cancer tissues. **f**, **g** The mRNA levels of IDO1 (**f**) increased, while miR-218 decreased (**g**) in HeLa, SiHa, C-33, and Caski cervical cells. **h**, **i** The protein levels of IDO1 increased in HeLa, SiHa, C-33, and Caski cervical cells. **j** The target sequences of miR-218 in the 3′-UTR fragment of IDO1 were identified by TargetScan bioinformatics analysis. **k** The co-transfection of miR-218 and IDO1-3′-UTR resulted in a significant downregulation of the luciferase activity. *P < 0.05 and **P < 0.01 vs. cervical epithelial HcerEpic cells in **f**–**i**. **P < 0.01 vs. Cntl+IDO1-3′-UTR group in **k**

RT-qPCR was used to detect the expression levels of miR-218 and IDO1 in 37 cervical cancer tissues. The results demonstrated that miR-218 levels were largely downregulated in cervical cancer tissues and that the survival rate of patients in the miR-218 low-expression group was lower than that in the miR-218 high-expression group within 2 years (Fig. 1a, b). Meanwhile, IDO1 levels were mostly upregulated in cervical cancer specimens, and the survival rate of patients in the IDO1 high-expression group was lower than that in the IDO1 low-expression group within 2 years (Fig. 1c, d). According to Spearman nonparametric correlation test (*P* = 0.0268, Fig. 1e), miR-218, and IDO1 were found to negatively correlate in cervical cancer tissues. The clinical pathological features were determined, and our results indicated that high IDO1 levels were correlated to advanced FIGO stages and low differentiated histological features of cervical cancer; however, it was not correlated with ages (Table 1).

Subsequently, we detected the mRNA expression levels of IDO1 and miR-218 in several cervical cancer cells such as HeLa, SiHa, C-33, and Caski cells. RT-qPCR results showed that the mRNA levels of IDO1 significantly increased, while miR-218 remarkably decreased in HeLa, SiHa, C-33, and Caski cervical cells, compared with cervical epithelial HcerEpic cells (*P* < 0.05, Fig. 1f, g). The protein levels of IDO1 were detected by Western blot, and the results showed similar pattern to that of the mRNA assay (*P* < 0.05, Fig. 1h, i).Expression changes of IDO1 and miR-218 were the greatest in HeLa cells among others. Therefore, HeLa cell line was used for miR-218 mimics transfection.

### MiR-218 directly targets the 3′-UTR fragment of IDO1

Using bioinformatics analysis of TargetScan, we found potential target sequences of miR-218 in the 3′-UTR fragment of IDO1 (Fig. 1j). Three bases in the target sequence were then mutated to obtain mutated 3′-UTR sequence of IDO1. Luciferase activity assay was used to observe the effect of miR-218 on the firefly luciferase activity of pmirGLO-UTR. The results showed that the co-transfection of miR-218 and IDO1-3′-UTR resulted in a significant downregulation of the luciferase activity of the reporter vector, and a statistically significant difference was identified compared to Cntl+IDO1-3′-UTR group (*P* < 0.01, Fig. 1k). However, no significant change in luciferase activity was found in the miR-218+IDO1-3′-UTR mut co-transfection group. The interaction between miR-218 and IDO1-3′-UTR affected the expression level of the reporter gene, suggesting that miR-218 might act on the 3′-UTR region of the IDO1 gene, regulating the expression of IDO1.

### MiR-218 overexpression inhibited cell viability and promoted cell apoptosis of cervical cancer cells

**Fig. 2:**
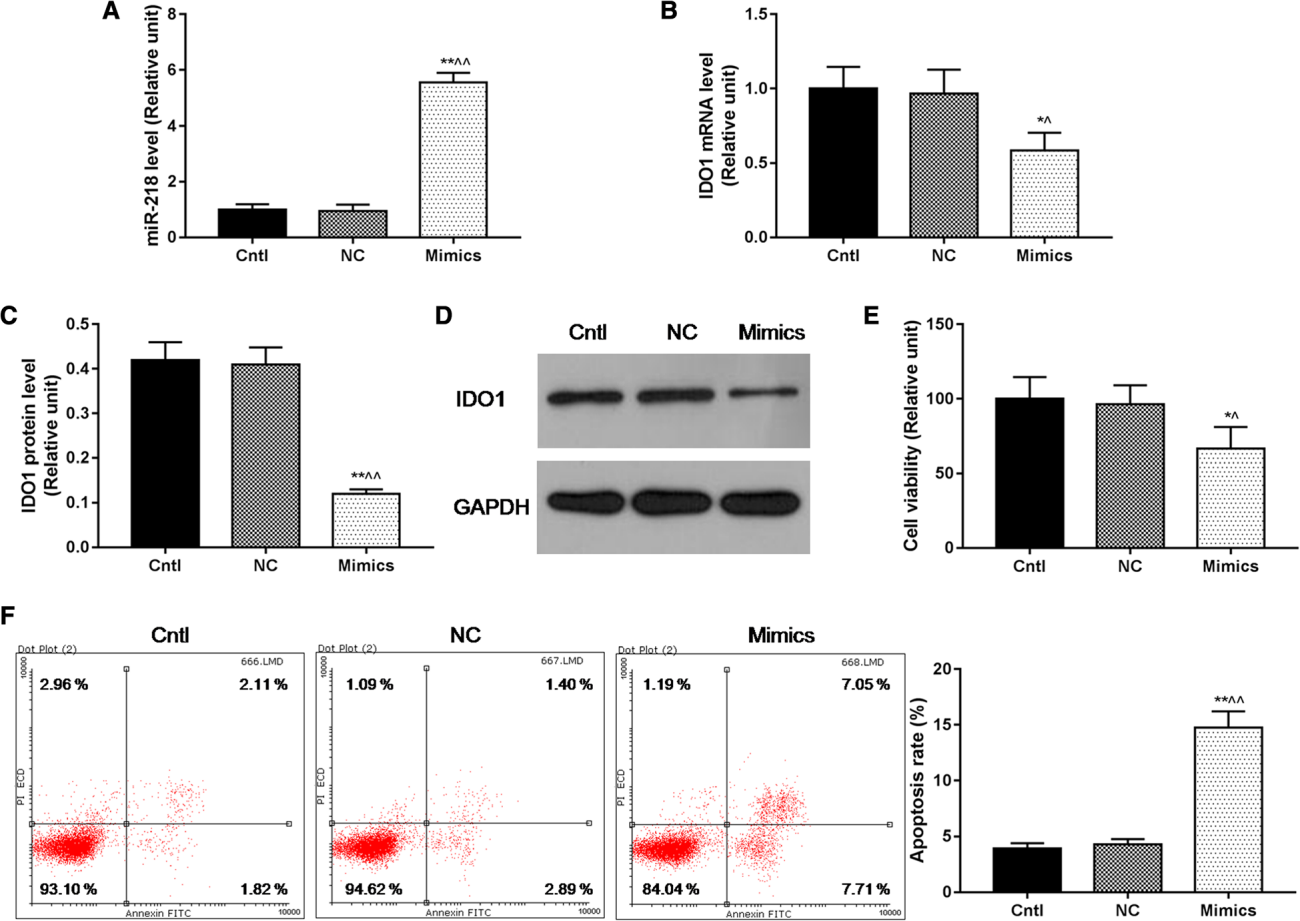
MiR-218 overexpression inhibited cell viability and promoted cell apoptosis of cervical cancer cells. **a** The mRNA levels of miR-218 were upregulated, in the Mimics group. **b–d** The mRNA (**b**) and protein (**c, d**) levels of IDO1 were downregulated in the Mimics group. **e**, **f** Cell viability (**e**) was inhibited, and apoptosis (**f**) was promoted in the Mimics group. *P < 0.05 and **P < 0.01 vs. the Cntl group, ^^^*P* < 0.05 and ^^^^*P* < 0.01 vs. the NC group

By performing RT-qPCR and Western blot assays (*P* < 0.05, Fig. 2a–d), we found that after miR-218 mimics has been transfected into cervical cancer HeLa cells, the mRNA levels of miR-218 were found to be sharply upregulated and that the mRNA and protein levels of IDO1 were markedly downregulated in the Mimics group, compared with the Cntl and NC groups. CCK-8 and Annexin-V/PI assays results showed that cell viability was significantly inhibited and that apoptosis was dramatically promoted in Mimics group, compared with the Cntl and NC groups (*P* < 0.05, Fig. 2e, f).

### MiR-218 overexpression inhibited cell viability and promoted cell apoptosis of cervical cancer cells by regulating apoptosis-related factors

**Fig. 3:**
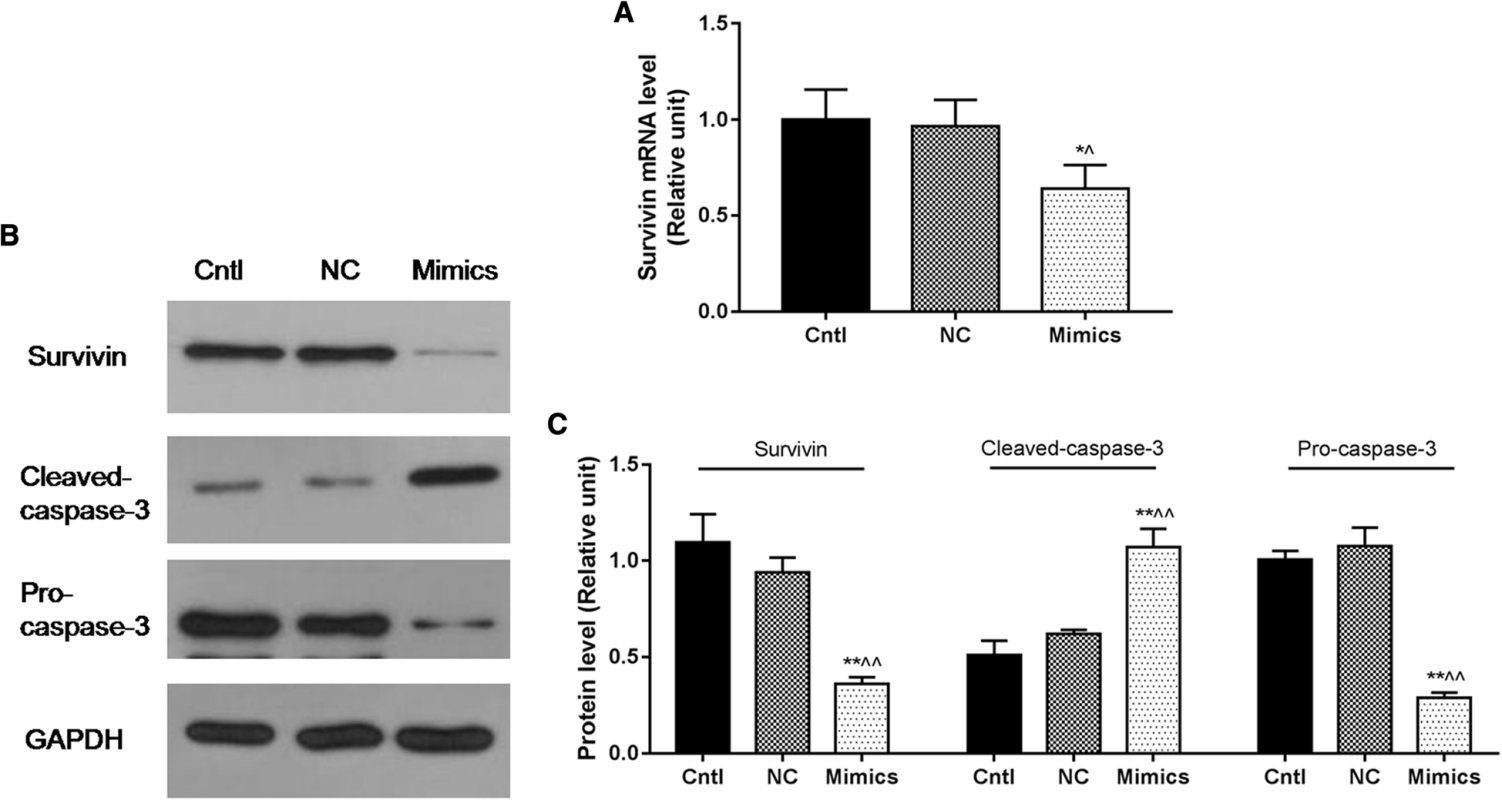
MiR-218 overexpression inhibited cell viability and promoted cell apoptosis of cervical cancer cells by regulating apoptosis-related factors. **a** The mRNA expression levels of Survivin was downregulated in the Mimics group. **b**, **c** The protein levels of Cleaved-Caspase-3 were upregulated and Pro-Caspase-3 was downregulated in the Mimics group. *P < 0.05 and **P < 0.01 vs. the Cntl group, ^^^*P* < 0.05 and ^^^^*P* < 0.01 vs. the NC group

As apoptosis is a mechanism that is characterized by some factors, we detected the expression levels of Survivin and Caspase-3. RT-qPCR showed that the mRNA expression levels of Survivin were downregulated, while the protein levels of Cleaved-Caspase-3 were upregulated and Pro-Caspase-3 was downregulated in the Mimics group, compared with the Cntl and NC groups (*P* < 0.05, Fig. 3).

### MiR-218 overexpression inhibited cell viability and promoted cell apoptosis of cervical cancer cells by regulating immune escape-related factors

**Fig. 4:**
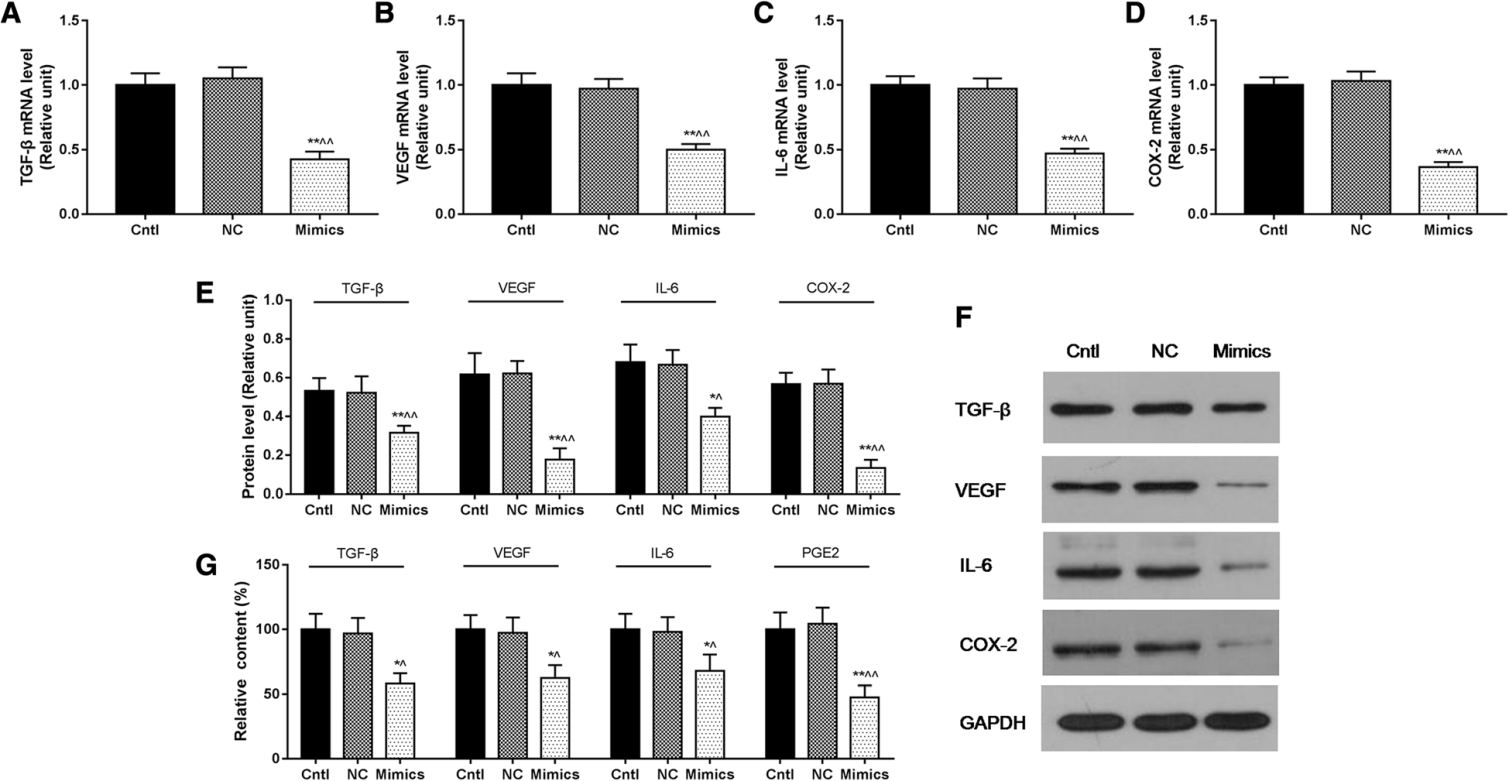
MiR-218 overexpression inhibited cell viability and promoted cell apoptosis of cervical cancer cells by regulating immune escape-related factors. **a–d** The mRNA levels of TGF-β, VEGF, IL-6, and COX-2 were significantly decreased in the Mimics group. **e**, **f** The protein levels of TGF-β, VEGF, IL-6, and COX-2 were significantly decreased in the Mimics group. **g** The relative content of TGF-β, VEGF, and IL-6 were significantly decreased in the Mimics group. *P < 0.05 and **P < 0.01 vs. Cntl group, ^^^*P* < 0.05 and ^^^^*P* < 0.01 vs. the NC group

Tumor immune escape can be characterized by levels of immune escape-related factors. In our study, RT-qPCR and Western blot were used to analyze the mRNA and protein levels of immune escape-related factors, for example, TGF-β, VEGF, IL-6, and COX-2, and they significantly decreased in the Mimics group (*P* < 0.05, Fig. 4a–f). ELISA was used to detect relative contents of TGF-β, VEGF, IL-6, and PGE2 to further verify the phenomenon, and the results showed significantly decreased levels of them (*P* < 0.05, Fig. 4g).

### MiR-218 overexpression inhibited cell viability and promoted cell apoptosis of cervical cancer cells via JAK2/STAT3 pathway

**Fig. 5:**
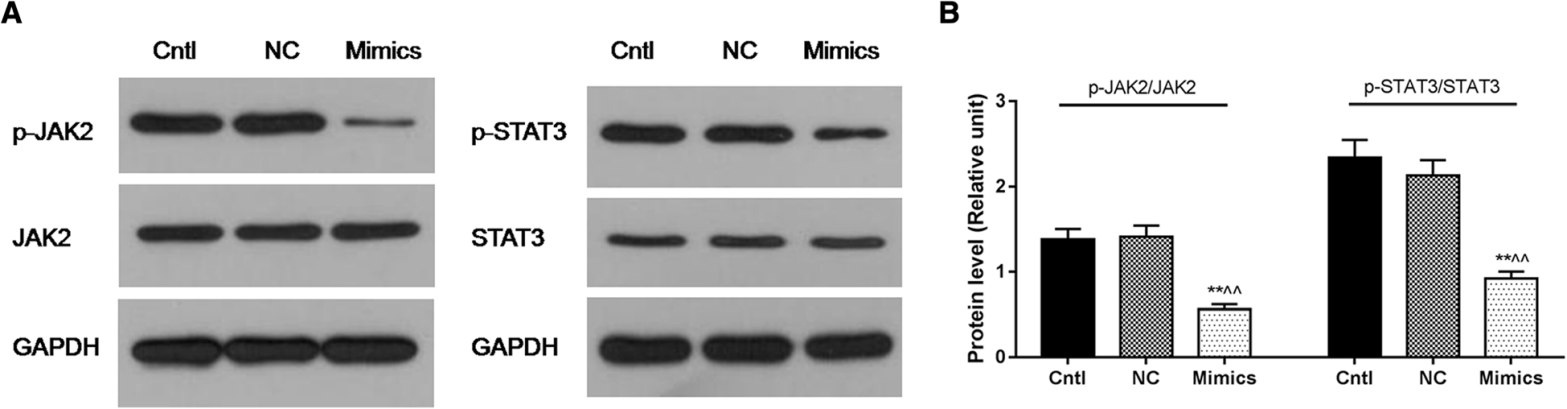
**a**, **b** MiR-218 overexpression inhibited cell viability and promoted cell apoptosis of cervical cancer cells via JAK2/STAT3 pathway. Less p-JAK2 and p-STAT3 in the Mimics group, compared with the Cntl and NC groups. **P < 0.01 vs. Cntl group, ^^^^*P* < 0.01 vs. the NC group

Western blot was performed to detect the activation status of JAK2 and STAT3. Our results observed less p-JAK2 and p-STAT3 in the Mimics group, compared with the Cntl and NC groups (*P* < 0.01, Fig. 5).

## Discussion

In tumor immune escape, IDO1 is a critically negative immunoregulatory factor, which induces the development, metastasis, and recurrence of tumors [32]. In this study, we adopted bioinformatics method and dual-luciferase reporter assay to confirm the direct targeting of miR-218 on the 3′-UTR of IDO1. Thus, miR-218 might have critical regulatory functions on the molecular mechanism of IDO1 in cervical cancer. Thirty-seven cervical cancer tissues were collected and detected, and we found that miR-218 was upregulated, while IDO1 was downregulated and that the two were negatively correlated with each other. In addition, high-level IDO1 and low-level miR-218 were correlated to advanced FIGO stages and low differentiated histological features of cervical cancers, as well as low survival rates in 2 years. Therefore, we studied the expression of miR-218 and IDO1 in some commonly used cervical cancer cells (HeLa, SiHa, C-33, and Caski cells), and the results showed the miR-218 was inhibited and the IDO1 levels were promoted. By transfecting miR-218 mimics into HeLa cervical cancer cells, we observed that the overexpression of miR-218 significantly suppressed the expression of IDO1, inhibited cell viability, and promoted apoptosis of cervical cancer cells.

Subsequently, variations of critical effectors in apoptosis caused by miR-218 overexpression were measured. Caspase-3, the direct execution factor to initiate apoptosis, is a cysteine protease protein specifically cleaving peptide bonds after aspartic acid residues. Caspase-3 usually acts as pro-caspase-3 in normal conditions; however, it will be phosphorylated and activated when apoptosis occurs. Survivin is a new member of the apoptotic inhibitor protein family, and it is also a tumor-specific apoptosis inhibitor that is expressed only in tumors and embryonic tissues. Survivin directly acts on Caspase by mainly inhibiting the activity of Caspase-3. In our study, miR-218 overexpression was observed to inhibit the expression of Survivin and promoted the activity of Caspase-3.

Researchers found that the body’s immune suppression was associated with a variety of cytokines [33]. TGF-β inhibits the immune system so as to protect tumor cells from being killed by immune cells [34]. The increase of VEGF levels in tumors reduces the immune function and promotes the formation of tumor interstitial blood vessels, allowing the tumors to grow rapidly [35, 36]. IL-6 stimulates the proliferation, differentiation, and function of cells involved in the immune response [37]. PGE2 is an important cell growth and regulation factor that can dilate blood vessels and can produce both immunosuppressive and anti-inflammatory effects. Under normal physiological conditions [38, 39], COX-2 is not expressed. However, in pathological conditions such as inflammation and tumors, COX-2 expression will be increased after being induced by pro-inflammatory mediators, for example, inflammatory stimulators, injuries, and carcinogens, participating in various pathological and physiological processes [40–42]. During our research, miR-218 overexpression inhibited the expression and function of these inflammatory factors TGF-β, VEGF, IL-6, PGE2, and COX-2 in cervical cancer cells.

JAK2/STAT3 signaling pathway and immune escape signaling pathway are important pathways of inflammatory response [43]. Many cytokines transfer from the outside to the nucleus via JAK2/STAT3 pathway and mediate the response of cells under the corresponding conditions [44]. STAT3 can mediate the onset of inflammatory reactions via IL-6/JAK pathway [45]. In addition, STATs can also modulate inflammatory/immune responses by regulating COX-2 and IDO, becoming intermediate bridges linking tumors and inflammatory responses [46]. In our study, the activation of JAK2 and STAT3 were significantly inhibited by miR-218 overexpression.

## Conclusions

. The expression of miR-218 was negatively correlated with IDO1 in cervical cancer. IDO1 is a direct target of miR-218. Overexpression of miR-218 exerted anti-tumor effects on cervical cancer through promoting apoptosis and inhibiting the expression of inflammatory factors. The inhibition of JAK2/STAT3 signaling pathway contributed to the anti-tumor effect produced by miR-218. This study will provide effective solutions for biological treatment of cervical cancer and other tumors.
